# Nutritional Strategies to Modulate Intracellular and Extracellular Buffering Capacity During High-Intensity Exercise

**DOI:** 10.1007/s40279-015-0397-5

**Published:** 2015-11-09

**Authors:** Antonio Herbert Lancha Junior, Vitor de Salles Painelli, Bryan Saunders, Guilherme Giannini Artioli

**Affiliations:** Laboratory of Applied Nutrition and Metabolism, School of Physical Education and Sport, University of São Paulo, Av. Mello de Moraes, 65 Butanta, São Paulo, SP 05508-030 Brazil

## Abstract

Intramuscular acidosis is a contributing factor to fatigue during high-intensity exercise. Many nutritional strategies aiming to increase intra- and extracellular buffering capacity have been investigated. Among these, supplementation of beta-alanine (~3–6.4 g/day for 4 weeks or longer), the rate-limiting factor to the intramuscular synthesis of carnosine (i.e. an intracellular buffer), has been shown to result in positive effects on exercise performance in which acidosis is a contributing factor to fatigue. Furthermore, sodium bicarbonate, sodium citrate and sodium/calcium lactate supplementation have been employed in an attempt to increase the extracellular buffering capacity. Although all attempts have increased blood bicarbonate concentrations, evidence indicates that sodium bicarbonate (0.3 g/kg body mass) is the most effective in improving high-intensity exercise performance. The evidence supporting the ergogenic effects of sodium citrate and lactate remain weak. These nutritional strategies are not without side effects, as gastrointestinal distress is often associated with the effective doses of sodium bicarbonate, sodium citrate and calcium lactate. Similarly, paresthesia (i.e. tingling sensation of the skin) is currently the only known side effect associated with beta-alanine supplementation, and it is caused by the acute elevation in plasma beta-alanine concentration after a single dose of beta-alanine. Finally, the co-supplementation of beta-alanine and sodium bicarbonate may result in additive ergogenic gains during high-intensity exercise, although studies are required to investigate this combination in a wide range of sports.

## Introduction

High-intensity exercise requires maximal or near-maximal intensity efforts resulting in rapid changes in the intramuscular metabolic profile. These changes include substrate depletion [1] and metabolite accumulation and are accompanied by muscular fatigue [2]. Exercise-induced muscle fatigue, defined as the inability of the skeletal muscle to maintain a particular tension or a given exercise intensity [3], has been a focal point of research for many decades. However, the exact mechanisms that contribute to fatigue remain poorly understood; fatigue is a complex and multifactorial phenomenon that varies depending on the type, intensity and duration of the exercise. In the particular case of high-intensity short-duration exercise, several contributing factors appear to be of particular concern to the onset of muscle fatigue, including the accumulation of potassium ions (K^+^) in the interstitium of the muscle cell [4], decreased release/uptake of calcium ions (Ca^2+^) from/to the sarcoplasmic reticulum [5], the depletion of energy substrates, and the accumulation of metabolites within the muscle cell [6].

Metabolite accumulation has long been considered one of the factors contributing to reduced exercise performance and capacity with the accumulation of hydrogen ions (H^+^), which causes acidification in the muscle, associated with muscle fatigue [2, 3, 7–10]. Analyses of muscle samples have consistently shown that pH values can decline from ~7.1 (at rest) to ~6.5 following high-intensity exercise to exhaustion [11–13]. The role of pH and the exact physiological mechanisms leading to fatigue remain controversial and are still under intense debate and investigation. Nonetheless, there is evidence to support the following roles of muscle acidosis in fatigue development: (1) competition of H^+^ with Ca^2+^ ions for the troponin binding site, impairing the ability of the contractile machinery to effectively operate [14, 15]; (2) inhibition of phosphorylcreatine resynthesis [16]; and (3) inhibition of key enzymes of the glycolytic pathway, such as glycogen phosphorylase and phosphofructokinase [17]. These effects may limit the ability of the muscle cells to cope with the high energy demand during exercise and result in a reduction in intensity and/or performance or complete cessation of exercise.

**Fig. 1:**
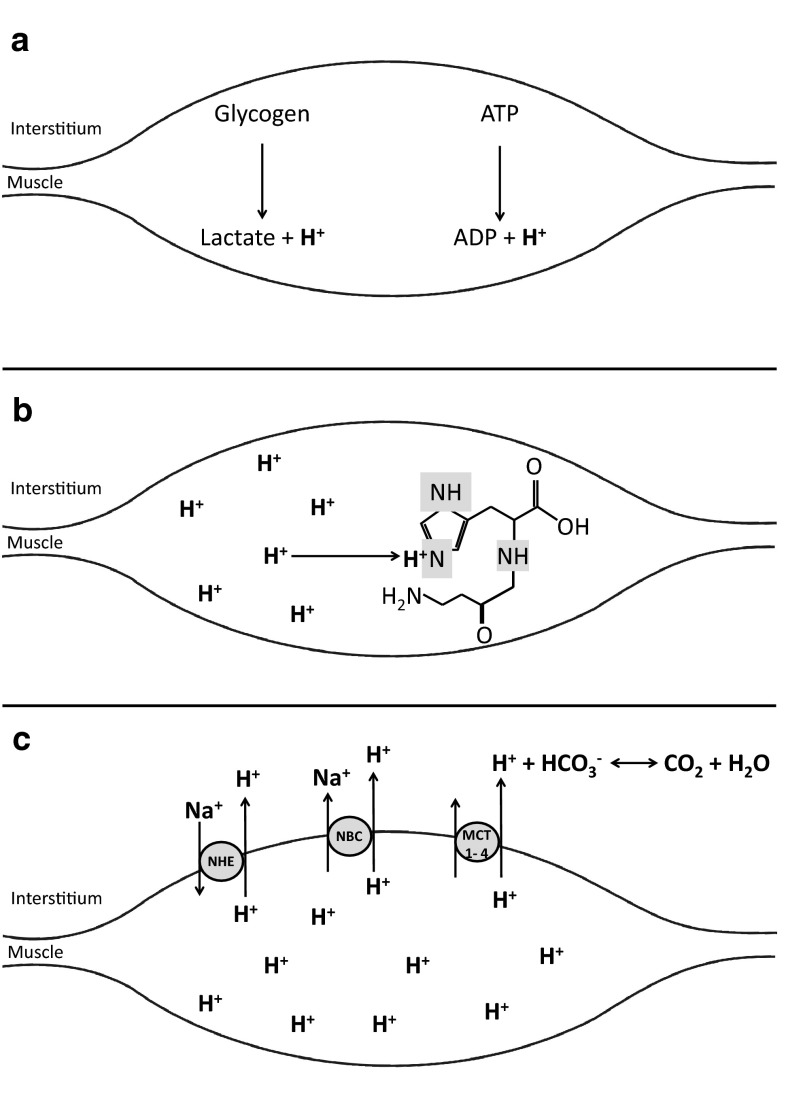
High-intensity exercise increases the energy demand of the muscle, which is met by aerobic and anaerobic energy sources. **a** The primary contributions of ATP degradation and anaerobic glycolysis to the production of H^+^ during exercise. Physico-chemical buffers (e.g. carnosine) represent the first line of defence against changes in muscle pH, and are the only defence during exercise when blood flow is occluded. **b** The carnosine molecule with its imidazole side chain where the accumulating H^+^ are buffered. In addition to intracellular buffering, transmembrane H^+^ transport is a major controller of pH during dynamic exercise. **c** The main transporters, including the sodium–hydrogen exchanger (NHE), the sodium bicarbonate co-transporter (NBC) and the monocarboxlate transporter (MCT1, MCT4). The circulating H^+^ are subsequently buffered by bicarbonate anions. *ADP* adenosine diphosphate, *ATP* adenosine triphosphate

The human body contains well-regulated mechanisms to maintain the intracellular and extracellular pH within the physiological range, including intracellular buffers, extracellular buffers, dynamic buffering systems, as well as respiratory and renal mechanisms for pH regulation [18, 19]. During high-intensity exercise, acid–base balance in muscle is mainly regulated by intracellular, extracellular and dynamic buffering (Fig. 1). Intracellular physicochemical buffering represents the immediate defence against the accumulation of H^+^ in the contracting muscle. This is mediated primarily by phosphates, proteins and dipeptides, which exert their buffering action in the cytosol, where pH is closer to the acid dissociation constant (Ka) of these substances. Muscle pH homeostasis is also regulated by active and passive transport of H^+^ into the surrounding interstitium, where they are buffered by circulating buffers, pulmonary ventilation and the kidneys. The flux of H^+^ out of the muscle during exercise is facilitated by MCT1 and MCT4, monocarboxylate transport proteins that carry monocarboxylates (i.e. lactate) across cell membranes, as well as by other transporting systems such as the sodium–hydrogen exchanger and the sodium bicarbonate co-transporter. In the blood, the chemical buffering system is primarily composed of bicarbonate (HCO_3_^−^), which has the ability to bind H^+^ [20].

The importance of the buffering systems in combating fatigue has led to increasing interest in nutritional strategies capable of increasing both intracellular and extracellular buffering capacity. This has led to a number of original investigations on a variety of supplements, including beta-alanine, sodium bicarbonate, sodium citrate, sodium and calcium lactate, with several narrative, systematic and meta-analytic reviews published on these topics [21–27]. The current review aims to summarise the available literature on all nutritional strategies aimed at increasing buffering capacity in light of more recent evidence and to highlight their underlying physiological mechanisms of action, effects on high-intensity exercise capacity and performance, and possible side effects.

## Beta-Alanine

### Mechanisms of Action

Beta-alanine is a non-essential and non-proteinogenic amino acid produced endogenously in the liver from the degradation of uracil [28]. Alternative synthesis from pathways in the gut [29] and kidney [30] might also account for some endogenous production of beta-alanine, although the very low fasting concentrations in blood [31] suggest that endogenous synthesis is rather low and does not constitute a significant source of beta-alanine for the tissues. Beta-alanine and l-histidine are the precursors of carnosine synthesis in skeletal muscle, a reaction catalysed by the enzyme carnosine synthase [32, 33].

Carnosine is a cytoplasmic dipeptide abundantly found in excitable tissues, such as skeletal muscle [31], heart [34] and in some brain regions [35], although the highest concentrations of carnosine in humans are found in skeletal muscle. Several physiological functions have been attributed to carnosine in skeletal muscle, including antioxidant activity [36] and protection against protein glycation and carbonylation [37, 38]. Data from in-vitro studies with animal and human muscle fibres have also attributed other functions to carnosine, including calcium sensitising, regulation of the calcium transient (i.e. increased calcium release and reuptake from the sarcoplasmic reticulum), and excitation-contraction coupling [37, 39]. However, a recent whole-body study with humans did not support the hypothesis of increased carnosine to increase calcium sensitivity and calcium release, but supported the finding that carnosine may improve calcium reuptake [40]. Clearly, more studies investigating these issues are still required to clarify the physiological roles of carnosine. Despite some controversy, an undisputed function of carnosine is intracellular pH regulation [31], since its side chain (i.e. the imidazole ring) has a pKa of 6.83 [41, 42], making carnosine an obligatory physicochemical buffer. Since the pKa of carnosine is optimal for buffering within the muscle during high-intensity exercise, it could be an important contributor to performance and tolerance to exercises that are limited by H^+^ accumulation.

Carnosine synthase, the enzyme responsible for carnosine synthesis in skeletal muscle, has a greater affinity, as indicated by its Michaelis–Menten constant (*K*_m_), for l-histidine (*K*_m_ ~16.8 µmol/L) than for beta-alanine (*K*_m_ ~1–2.3 mmol/L) [43, 44]. Moreover, plasma and intramuscular concentrations of l-histidine are substantially higher than beta-alanine [45]. Consequently, beta-alanine availability is the rate-limiting factor for the endogenous synthesis of carnosine within skeletal muscle. Subsequently, beta-alanine supplementation (over a period of 2 weeks or longer) induces significant increases in muscle carnosine content [31, 46, 47]. The typical increase in muscle carnosine following beta-alanine supplementation is 60–80 %, which is estimated to elevate the contribution of carnosine to whole muscle H^+^ buffering by ~2.7–5.3 mEq H^+^/kg dry mass over the exercise pH range [31], corresponding to an ~3–5 % increase in muscle buffering capacity. Although this is a conservative estimate of the contribution to the whole muscle, the specific contribution to the buffering capacity of type II fibres is considerably greater. Theoretically, such an increase in intracellular buffering capacity could translate into concomitant increases in performance and capacity during exercise limited by increasing muscle acidosis.

### Effects on High-Intensity Exercise

A meta-analysis of the available evidence on beta-alanine and exercise [24] showed that beta-alanine improved exercise to a greater extent than placebo. The positive effect was due to improvements in exercise capacity tests but not performance tests, though this may have been due to the relatively low number of performance studies at the time of analysis. Since a capacity test requires the individual to exert to the point of volitional exhaustion, as opposed to a fixed point of cessation in a performance test, thereby resulting in a maximal production of H^+^, these data support the role of carnosine in acid–base balance. A highly significant effect was shown for exercises lasting 60–240 s, which strengthens the suggestion that the primary role of muscle carnosine is pH buffering. With the growing popularity of beta-alanine, numerous investigations have been published since this meta-analysis and have shed further light on the ergogenic potential of this nutritional supplement.

There is clear evidence that exercises shorter than 60 s are unaffected by beta-alanine [48–50]. This consistent outcome contrasts the purported role of increased carnosine to increase the calcium sensitivity of the muscle and the calcium release from sarcoplasmic reticulum. This was confirmed by Hannah et al. [40], who showed no effect of beta-alanine on maximum and explosive voluntary contractions, but there was a reduction in half-relaxation time, suggesting an enhanced reuptake of calcium. Further mechanistic studies are required to determine the role of carnosine in calcium handling.

Several studies have shown the efficacy of increased muscle carnosine content through beta-alanine supplementation on high-intensity exercises lasting 1–4 min; exercise performance and capacity have been improved in a variety of cycling [46, 51–54], running [55, 56], and repeated-bout upper- and lower-body protocols [57, 58]. The majority of research reporting an ergogenic effect of beta-alanine supplementation is on exercise lasting 1–10 min, although not all agree [59–62]. Improvements in high-intensity cycling capacity at 110 % of maximum power output, a reliable cycling capacity test designed to last between 120–240 s [63], have consistently been shown, with improvements of 11.9 % [46], 12.1 % [54] and 14.0 % [64] in time-to-exhaustion in recreationally active participants following beta-alanine supplementation. These studies highlight the consistency in responses across individuals following supplementation during a high-intensity test limited by increasing acidosis.

Hobson et al. [24] showed an effect of beta-alanine on exercise lasting more than 240 s, although this result was likely due to the incremental nature of many of the tests employed, which are very low in intensity in the earlier stages [51, 52]. Two studies did not show any improvement on prolonged cycling time trial performance [53, 62], which is in line with the role of carnosine as an intramuscular buffer since fatigue during exercise of this duration is not associated with increasing acidosis.

Some doubt has been raised concerning the efficacy of β-alanine on athletes since sprint-trained individuals have been shown to have an elevated buffering capacity compared with their non-trained and endurance-trained counterparts [65, 66]. It has been argued that previously elevated muscle buffering capacity could minimise any improvements brought about via increased carnosine. This has gained some support from indirect evidence in studies with well-trained athletes who supplemented with beta-alanine and showed no improvements in performance [48, 50, 59, 60]. However, a recent study in our laboratory, specifically designed to address this topic, investigated the effects of beta-alanine supplementation on high-intensity cycling performance in both trained cyclists and non-trained individuals. Both groups showed a similar improvement in total work done (~3 %) following supplementation [58], indicating that beta-alanine is equally efficient in untrained and athletic populations. Indeed, there is now a growing body of evidence to support the effective use of beta-alanine among the elite athletic populations; athletes involved in 100- and 200-m freestyle swimming [67], 2000-m rowing [68–70] and 800-m running [55].

### Possible Side Effects

The only currently known side effect reported in the literature from the use of beta-alanine is paraesthesia, which has been described as a prickly sensation on the skin that starts within 10–20 min following ingestion and lasts up to 1 h [31]. These symptoms typically arise from high doses of beta-alanine and are associated with the peak plasma beta-alanine level [31]. It has been suggested that beta-alanine stimulates a specific G-protein-coupled receptor expressed by sensory neurons located at the surface of the skin [71]. Although harmless, paraesthesia is unpleasant and may compromise the blinding of a research investigation; therefore, dosing strategies aiming to avoid paraesthesia are employed. To circumvent the occurrence of paraesthesia, studies have consistently staggered dosing protocols throughout the day. Harris et al. [31] were successful in reducing the incidence of paraesthesia with multiple doses of 800 mg every 3 h. A new sustained-release formulation has been developed that results in a lower peak plasma concentration from a single dose while release into blood and uptake into muscle is maintained over 6 h with minimal side effects [72].

In light of the current evidence suggesting no major side effects other than paraesthesia during supplementation of 2–16 weeks in duration [46, 47, 73], athletes can safely supplement with beta-alanine for this period of time prior to competition or throughout training. Future research should ascertain as to whether longer-term supplementation (>16 weeks) is free of any other side effects, although the preliminary results of a study we are currently conducting showed that beta-alanine supplementation did not alter clinical markers of health in 16 subjects who supplemented for 6 months.

## Sodium Bicarbonate

### Mechanisms of Action

Bicarbonate is a blood buffer that plays an important role in maintaining both extracellular and intracellular pH, despite its inability to permeate the sarcolemma [12, 74]. Under normal resting conditions, circulating concentrations of bicarbonate range between 23 and 27 mmol/L [75]. Studies investigating acute sodium bicarbonate supplementation to increase blood bicarbonate levels have used doses relative to body mass (BM), ranging from as little as 0.1 g/kg, increasing to as much as 0.5 g/kg BM [76]. Sodium bicarbonate supplementation has consistently caused blood alkalosis [12, 77, 78] accompanied by an increase in blood bicarbonate concentrations (for a review, see Carr et al. [22]).

As a consequence of increased blood pH and HCO_3_^−^, there is a parallel increase in the intracellular/extracellular gradient of H^+^, enhancing the activity of the lactate/H^+^ co-transporters [79–81], ultimately leading to a greater efflux of H^+^ and lactate out of the active muscles and into the circulation, where they are buffered or can be taken up into adjacent [82] or inactive [83] muscle fibres. Therefore, sodium bicarbonate ingestion increases the extracellular buffering and dynamic buffering capacity, increasing the rate at which accumulating H^+^ is removed from the working muscles during high-intensity exercise, which ultimately contributes to intramuscular pH maintenance. An attenuation of H^+^ accumulation in the working muscle would allow the contractile process and the resynthesis of adenosine triphosphate (ATP) by glycolysis to continue under more favourable conditions, thereby delaying the onset of muscle fatigue during high-intensity exercise.

### Effects on High-Intensity Exercise

The effects of sodium bicarbonate supplementation on exercise performance and capacity have been well researched. In a meta-analysis of the literature, Carr et al. [22] showed that sodium bicarbonate was effective at improving a 1-min all-out bout by 1.7 ± 2.0 % (mean ± 95 % confidence limits) when ingested at a dose of 0.3 g/kg BM prior to exercise. The ergogenic effects of sodium bicarbonate were enhanced with increasing doses and sprint bouts, though a dose of 0.3 g/kg BM is generally considered optimal since McNaughton [76] showed no further improvements in 60 s of cycling with increasing doses.

An improvement of 1.7 % in performance may appear to be minor, but it is important to highlight the discrepancy between studies that have contributed to inconsistent results. Methodological differences such as differing dosing regimens, exercise types and intensities as well as side effects and individual variation have likely contributed to differing performance improvements, potentially masking the true magnitude of the performance effect of sodium bicarbonate. Saunders et al. [84] showed a large inter-individual ergogenic response to sodium bicarbonate while also highlighting the negative impact that the associated side effects can have on performance. It also has been suggested that general analyses of intervention studies do not account for a potentially high variation within individuals which may compromise the magnitude of effect of an intervention. Therefore, caution should be exercised when interpreting mean and pooled data regarding the efficacy of sodium bicarbonate as an ergogenic aid.

However, it is important to highlight several individual studies, particularly those employing multiple bouts of supramaximal exercise, since exercise of this type may elicit higher muscle acidosis than continuous supramaximal exercise [85, 86]. Studies using multiple bouts of high-intensity exercise have consistently shown performance improvements in excess of 8 % [57, 87, 88]. In addition, there is evidence to suggest that sodium bicarbonate is also beneficial to sport-specific performance in a variety of disciplines where the metabolic demands are predominantly anaerobic, such as judo, swimming, boxing and water polo [89–92]. This also suggests that the ergogenic effects observed in exercise capacity tests can be translated into performance improvements in real sport settings.

### Possible Side Effects

The ergogenic potential of sodium bicarbonate appears to be highly variable, with acute gastrointestinal side effects associated with supplementation a possible confounding factor that may preclude improvements in performance [84]. Ingestion of sodium bicarbonate results in the release of HCO_3_^−^ within the stomach, which is swiftly neutralised by the H^+^ found in the stomach’s gastric juice, which leads to increased CO_2_ production, and may cause abdominal pain, flatulence, nausea, diarrhoea and even vomiting [93]. Incidence and severity of symptoms differ between individuals [65] and may also be influenced by dose, as McNaughton [76] reported increased gastrointestinal disturbance in all participants consuming doses above 0.3 g/kg BM.

Several strategies have been adopted in order to minimise the discomfort associated with acute sodium bicarbonate supplementation, including multiday ingestion [94], chronic administration [95] and split-dose protocols [54, 96]. However, the large doses used in chronic supplementation protocols (normally 500 mg/kg/day) present other obstacles that may prevent athletes from taking sodium bicarbonate, such as the excessive amount of sodium ingested over a relatively long period and the continuous sensation of satiety due to the excessive number of capsules. Furthermore, split-dose strategies may still lead individual to experience severe gastrointestinal discomfort [84]. Future research should try to solve the challenges inherent in the use of this nutritional supplement, focusing on alternatives that minimise the dose and/or attenuate the side effects associated with sodium bicarbonate supplementation. Athletes should engage in supplementation during pre-season or training to determine their tolerance to the supplement.

## Sodium Citrate

### Mechanisms of Action

Sodium citrate is another agent capable of increasing extracellular buffering capacity. Upon ingestion, it is rapidly dissociated to its constituent ions; the citrate anion is expelled from the plasma, leading to a change in the electrical equilibrium [97, 98]. In order to recover this equilibrium, a decrease of H^+^ in plasma occurs as well as a resultant increase in HCO_3_^−^, enhancing the extracellular buffering capacity [99].

The increase in blood pH through sodium citrate supplementation facilitates a greater efflux of H^+^ and lactate from the active muscles via monocarboxylate transporter proteins that carry monocarboxylates across the cell membrane [100]. Potteiger et al. [101] showed peak values of pH and HCO_3_^−^ in blood 100–120 min following the ingestion of 0.5 g/kg BM of sodium citrate, suggesting this timing strategy may be most effective in increasing extracellular buffering capacity, though the most effective dose remains yet to be elucidated.

### Effects on High-Intensity Exercise

The effects of sodium citrate on exercise have been well documented, with numerous original investigations assessing its ergogenic capacity [97–112]. However, the results are inconsistent, as revealed by a meta-analysis showing an unclear effect on performance of 0.0 ± 1.3 % [22]. Early studies investigating the potential ergogenic effects of this nutritional strategy did not show any positive effects of 0.3 g/kg BM sodium citrate on a variety of exercise protocols [97, 98, 102]. Later, McNaughton [103] showed that 0.5 g/kg sodium citrate was a more favourable dose to optimise anaerobic performance. Indeed, McNaughton and Cedaro [99] demonstrated that this dose of sodium citrate significantly improved high-intensity cycling lasting 120–240 s. In exercise protocols of shorter duration (i.e. 10- and 30-s cycling protocols), performance was not improved by an equivalent dose, likely because buffering capacity is not critical to performance in such short protocols. The ergogenic effect of sodium citrate on high-intensity cycling 1–4 min in duration has been further demonstrated using a supramaximal cycling test [102–104].

The effects of sodium citrate on running appears more uncertain, with a 0.5 g/kg BM dose showing only a 50 % likelihood of an improvement in an all-out sprint to exhaustion in endurance athletes [105]. Further sport-specific running protocols have provided more equivocal results. A low dose of 0.3 g/kg did not improve 600-m running performance in female athletes and non-athletes [106], whereas a higher dose of 0.5 g/kg did improve 3000-m running performance in male and female athletes [107] and 5000-m running performance in well-trained college runners [108]. However, further research from this group has yielded contrasting evidence with no effect of an equivalent dose on 5-km time trial in trained male runners [109] or 1500-m performance in trained female middle-distance runners [110] following 0.4 g/kg sodium citrate. Furthermore, sodium citrate was equally unable to improve repeated 60-s sprints in moderately active males [111].

Assessment of the literature reveals a variable exercise response following sodium citrate supplementation, with an apparent increased efficacy during cycling versus running protocols. Isolated muscle groups may be more susceptible to local acidosis than whole body exercise. This may explain the lower effectiveness of increased buffering capacity on running as compared with cycling protocols. Nonetheless, caution should be exercised when interpreting these results because of differences in supplementation doses. Furthermore, the absence of well-controlled familiarisation sessions and standardisation of the gender and training status within a study cannot be ruled out as possible factors leading to inconsistency in results. Further studies should be conducted covering all these limitations in order to better evaluate the true ergogenic efficacy of sodium citrate.

### Possible Side Effects

Sodium citrate is frequently employed as an alternative to sodium bicarbonate because of the common belief that it results in decreased side effects. Despite this, individuals have reported thirst, nausea and headaches following ingestion of 0.5 g/kg BM sodium citrate, with all incidences occurring within the first 60 min post-ingestion [103]. Similarly, several participants reported symptoms of gastrointestinal discomfort and stomach cramps following 0.5 g/kg [112] and 0.6 g/kg [109] of sodium citrate, although not all participants in these studies reported discomfort. However, there is sufficient evidence to suggest that the ingestion of sodium citrate may cause discomfort that may negate any performance benefits through increased buffering capacity. The individual variability suggests that the efficacy of this strategy should be individually tested by athletes in training prior to actual competitions.

## Sodium Lactate and Calcium Lactate

### Mechanisms of Action

Lactate supplementation has been suggested as a strategy capable of increasing extracellular buffering capacity [105, 113]. The lactate ingested is absorbed preferentially at the jejunum, through the sodium-coupled intestinal lactate transporter (sMCT), also known as the SLC5A8 [114–116]. Once in the bloodstream, it can be taken up by several tissues, including skeletal muscle, where it is oxidised [117]. Alternatively, lactate can be taken up by hepatocytes, where it is converted into glucose [118]. In both reactions, there is a net consumption of H^+^, which could indirectly spare blood bicarbonate and increase blood pH [113, 119], increasing lactate and H^+^ efflux from the muscle, therefore decreasing intramuscular H^+^ concentration [79, 80].

Research has investigated both the effects of sodium lactate and calcium lactate, though it is currently unknown how they differ in terms of their alkalosis-inducing effects. Due to the sodium-coupled transport of lactate at the jejunum [114–116], one might speculate that sodium lactate would be absorbed faster than calcium lactate. The fact is that there are currently no dose-response studies on oral sodium lactate ingestion or directly comparing blood pH and HCO_3_^−^ responses between calcium and sodium lactate. However, data from our laboratory showed that the ingestion of 150 and 300 mg/kg BM of calcium lactate induced a small, yet significant, increase in blood pH and HCO_3_^−^ [120], with peak values being attained 90 min post-ingestion. Interestingly, there were no differences in blood pH and HCO_3_^−^ following the different doses, suggesting that 150 mg/kg may be sufficient, though further research is needed.

### Effects on High-Intensity Exercise

Since the use of lactate supplementation as a buffering agent is relatively novel, the available evidence of its ergogenic potential is scarce and controversial. Van Montfoort et al. [105] were the first to investigate the buffering efficacy of lactate, supplementing individuals with 400 mg/kg BM sodium lactate, with participants run to exhaustion 90 min post-ingestion. Exercise capacity was improved with sodium lactate by 1.7 % compared with placebo, with an 83 % likelihood of the difference being meaningful. Subsequently, Morris et al. [113] supplemented trained cyclists with 120 mg/kg calcium lactate 80 min prior to exercise and showed a performance improvement of 17 % compared with placebo during a cycle-to-exhaustion protocol following a supramaximal multiple-bout protocol. Although the results of both studies are encouraging, the discrepancy between doses and subsequent exercise improvements suggested that further research is required.

In order to gain further insight into this emerging supplement, our research group investigated the acute effects of two different dosages of calcium lactate (150 and 300 mg/kg BM) on high-intensity intermittent performance in the form of three upper-body arm-crank bouts (this protocol has previously been shown to be sufficiently sensitive to detect performance changes due to increased buffering capacity) [57, 58, 89]. Despite significant increases in blood pH and HCO_3_^−^, no improvements in performance were shown with either dose [120]. These data have cast some doubt and further controversy on the efficacy of lactate supplementation as the dosing protocols are very similar between all these studies. A possible explanation for the beneficial effects of lactate supplementation in the previous studies, but not in ours, would be related to the exercise protocols. Van Montfoort et al. [105] and Morris et al. [113] employed lower-body time-to-exhaustion protocols, and capacity tests are generally less reliable than performance tests and have been known to have coefficients of variation in excess of 10 % [121]. Furthermore, the repeated-bout Wingate-like test employed by our group was an intermittent upper-body performance test, which results in even greater acidosis than leg cycling exercises [122], and theoretically would make our protocol more sensitive to detect the effects of calcium lactate ingestion on performance. In view of this, future studies should investigate different exercise performance protocols and different supplementation protocols, such as chronic administration of lactate, which could result in a more pronounced blood alkalosis.

### Possible Side Effects

The only reported side effects from sodium/calcium lactate supplementation resulted from a recent study conducted in our laboratory [120]. Low- and high-dose calcium lactate supplementation induced eructation and flatulence to a similar extent. No side effects were reported in other studies [105, 115]. Though initial reports appear favourable, only further research will determine whether different doses and types of lactate supplementation will result in any detrimental side effects.

## Co-Supplementation

### Mechanisms of Action

The buffering systems of the human body are complex and work simultaneously to maintain intracellular and extracellular homeostasis. Since these systems do not work independently, it could be hypothesised that an increase in the capacity of multiple buffering systems would lead to a better improvement in pH regulation. In line with this, several studies have investigated the effects of co-supplementation of buffering agents. However, since sodium bicarbonate, sodium citrate and sodium/calcium lactate result in the same physiological response (i.e. increased blood pH and HCO_3_^−^), studies on co-supplementation are limited to the use of a buffering agent capable of increasing extracellular buffering capacity combined with beta-alanine (capable of increasing intracellular buffering capacity). This could lead to an increase in intracellular H^+^ buffering while simultaneously increasing the efflux of H^+^ out of the working muscle. Another limiting factor for the combination of bicarbonate, citrate and/or lactate is the high dose required, and given that one supplement may cause gastrointestinal disturbances, adding high doses of more than one of these alkalising agents is likely to be impractical.

### Effects on High-Intensity Exercise

Sale et al. [54] were the first to investigate the effect of co-supplementation of beta-alanine and sodium bicarbonate on high-intensity cycling capacity. Although null-hypothesis significance testing indicated there was no benefit of co-supplementation above beta-alanine alone, magnitude-based inferences showed a 70 % likelihood that the differences observed were meaningful. This is in contrast to the results of Bellinger et al. [59], where trained cyclists showed no further performance benefits in 4 min of cycling when co-supplemented. However, the cyclists in this study showed no performance gains with beta-alanine alone, but did with sodium bicarbonate. Since it was expected that increased buffering capacity would improve performance regardless of nutritional strategy, these results are somewhat surprising and test performance may have been influenced by pacing strategies. Further research on single-bout high-intensity exercise has suggested that co-supplementation may lead to small additive gains in 100- and 200-m swimming time-trial performance [67].

Repeated-bout high-intensity exercise may induce a greater severity of muscle acidosis, particularly in the latter bouts [86], leading to several studies that have investigated the effects of co-supplementation of beta-alanine and sodium bicarbonate on exercise of this type. No beneficial effects were shown on repeated bouts of <10 s in duration [96, 123]. However, clear additive gains were shown during four bouts of 30-s arm-cranking in trained grappling athletes [57]. Interestingly, there was an ~7 % improvement in total work done following supplementation with beta-alanine and sodium bicarbonate alone, and this was doubled to 14 % with co-supplementation. The clear additive effect of co-supplementation shown in this study is in contrast to previous research, though results may be due to the chronic sodium bicarbonate supplementation protocol employed in this study. However, pre-exercise blood pH and bicarbonate values were not determined, so it is unknown whether chronic supplementation resulted in greater blood alkalosis. Another explanation may be related to the exercise protocol used in this study. The four-bout upper-body test has been shown to elicit extreme metabolic acidosis, with blood pH values often reaching ~6.9–7.0. Since the muscle groups involved in the arm-crank test are relatively small, the elevated whole-body metabolic acidosis suggests a dramatic acidosis within the contracting muscles. Therefore, this test may provide the optimal conditions to determine the effects of enhanced buffering capacity.

Although evidence thus far is contradictory, there is currently a paucity of research on the co-supplementation of intracellular and extracellular buffering agents on exercise. The lack of clear effects may be due to a number of confounding factors including exercise protocols unaffected by increased buffering capacity and influenced by pacing strategies. Furthermore, the evidence regarding the efficacy of extracellular buffering agents appears much more variable than that of beta-alanine, with a number of further compounding factors contributing to this inconsistency.

### Possible Side Effects

None of the investigations that evaluated co-supplementation of beta-alanine and sodium bicarbonate reported any side effects outside of those already discussed for each supplement separately. Therefore, similar to previous recommendations, individuals should consume beta-alanine observing maximum single doses and daily doses, while monitoring their response to sodium bicarbonate during training or out of competition.

## Conclusions and Future Perspectives

The evidence to support the effectiveness of intracellular and extracellular buffers is substantial. Beta-alanine supplementation can increase intracellular buffering capacity through increased carnosine concentration. This includes compelling evidence highlighting its ergogenic potential across several exercise modalities and durations, specifically 1–10 min, and also within trained and non-trained populations. Importantly, the consistency of beta-alanine supplementation to improve exercise performance has been shown across individuals within the same exercise test [46, 54, 64]. Furthermore, current evidence suggests the side effects with beta-alanine supplementation to be minimal and manageable. Since a low dose (3.2 g/day) can increase muscle carnosine within 2 weeks [47] with chronic supplementation resulting in sustained increased buffering capacity over time, beta-alanine appears to be the most effective buffering agent to improve exercise capacity and performance during single- and repeated-bout high-intensity exercise.

The effects of increased extracellular buffering capacity on exercise appear more inconsistent despite the fact that there are three buffering agents capable of inducing blood alkalosis. This is likely due to a high degree of inter-individual variability, which may have masked the true magnitude of effect in studies employing a solitary intervention trial [124]. Future studies should perform repeated interventional trials with the same individuals to allow quantification of individual responses and consistency in responses. The study by Van Montfoort et al. [105], which directly compared these three buffering agents, showed that sodium bicarbonate was most effective in improving exercise performance, followed by sodium lactate, with sodium citrate being the least effective. Though the quality and quantity of research into sodium and calcium lactate remains incomplete, the authors believe this hierarchy of efficacy to be the most pertinent, though caution is recommended when ingesting these extracellular buffering supplements due to their associated side effects.
